# Reactivated cytomegalovirus proctitis in an immunocompetent patient presenting as nosocomial diarrhea: a case report and literature review

**DOI:** 10.1186/s12879-017-2218-y

**Published:** 2017-02-01

**Authors:** Chun-Yuan Lee, Yen-Hsu Chen, Po-Liang Lu

**Affiliations:** 1 Division of Infectious Diseases, Department of Internal Medicine, Kaohsiung Medical University, Kaohsiung Medical University Hospital, Kaohsiung, Taiwan; 2 Sepsis Research Center, Graduate Institute of Medicine, College of Medicine, Kaohsiung Medical University, Graduate Institute of Medicine, Kaohsiung, Taiwan; 3 School of Medicine, College of Medicine, Kaohsiung Medical University, Kaohsiung, Taiwan; 4 Department of Biological Science and Technology, College of Biological Science and Technology, National Chiao Tung University, Hsin Chu, Taiwan; 5 Department of Laboratory Medicine, Kaohsiung Medical University Hospital, Kaohsiung, Taiwan

**Keywords:** Case report, Cytomegalovirus, Immunocompetent, Proctitis

## Abstract

**Background:**

Reactivated cytomegalovirus (CMV) infection has been known to cause significant morbidity and mortality in immunocompromised patients. However, CMV disease rarely develops in immunocompetent patients, and reported cases often present with a mild, self-limiting course, without severe life-threatening sequelae. While the colon is the most common gastrointestinal site affected by CMV disease in immunocompetent patients, rectal involvement is rarely reported. CMV proctitis can present in two distinct forms, primary and reactivated. However, reactivated CMV proctitis is rarely reported as a causative etiology of nosocomial diarrhea, except in transplant patients. Herein we present a case of reactivated CMV proctitis in an immunocompetent patient, presenting as nosocomial diarrhea. Previously reported cases of reactivated CMV proctitis in immunocompetent patients are also reviewed.

**Case presentation:**

A 79-year-old female was admitted because of metabolic encephalopathy caused by dehydration and hypernatremia. The patient’s consciousness level returned rapidly after fluid supplementation. However, she subsequently presented with abdominal pain and diarrhea on day 8 of admission. Abdominal contrast-enhanced computed tomography on day 10 of admission demonstrated inflammation around the rectum, suggesting proctitis. Colonoscopy on day 16 of admission showed a giant ulcer at the rectum. Pathology of rectal biopsy confirmed CMV infection. The patient recovered without sequelae after 38 days of valganciclovir treatment. Follow-up colonoscopy revealed a healed ulcer over the rectum. Ten cases in the literature, plus our case, with reactivated CMV proctitis in immunocompetent patients were reviewed. We found that most patients were elderly (mean, 72 years) with a high prevalence of diabetes mellitus (54.5%). Cardinal manifestations are often non-specific (diarrhea, hematochezia, tenesmus), and eight (72.7%) developed CMV proctitis following a preceding acute, life-threatening disease, rather than as an initial presentation on admission. These manifestations frequently develop during hospitalization, and are thus often regarded as nosocomial diarrhea.

**Conclusions:**

Clinicians should be aware of the possibility of nosocomial onset of reactivated CMV proctitis in patients hospitalized due to a preceding critical illness, although the benefits of antiviral therapy remain unclear.

## Background

Cytomegalovirus (CMV) is a member of the Herpes virus family, and CMV disease can lead to significant morbidity and mortality in immunocompromised patients [1]. Through direct and indirect mechanisms, CMV can cause mononucleosis-like syndrome, tissue invasive disease, opportunistic infection, and post-transplant lymphoproliferative disorder in immunocompromised patients [1]. However, CMV disease rarely develops in immunocompetent patients, and reported cases often present with a mild, self-limiting course, without severe life-threatening sequelae [2].

A recent review of 290 cases of CMV disease in immunocompetent patients demonstrated the gastrointestinal (GI) tract to be the most commonly involved site, occurring in 91 patients (31%) [2]. While the colon is the site of the GI tract most commonly affected, the rectum is rarely involved [2]. CMV proctitis can present in two distinct forms: primary and reactivated [3]. Mononucleosis-like illness with rectal bleeding within several days to 2 weeks after unprotected anal intercourse in young patients is pathognomonic for primary CMV proctitis [3]. In contrast, reactivated CMV proctitis mainly occurs in elderly patients, without exposure to anal intercourse, who have multiple comorbidities, such as diabetes mellitus (DM), inflammatory bowel disease, and multi-organ failure [3]. However, CMV reactivation is rarely reported as a causative etiology of nosocomial diarrhea, except in transplant patients [4, 5].

We present here a case of reactivated CMV proctitis in an immunocompetent patient, presenting as nosocomial diarrhea, and previously reported cases of reactivated CMV proctitis in immunocompetent patients are also reviewed.

## Case presentation

A 79-year-old female with underlying diseases of DM, major depression disorder, and coronary heart disease was admitted because of altered mental status for 5 days. She could take care of herself until 5 days before admission. However, at this time, her family found that she could not recognize them. She had no fever, chills, or gastrointestinal discomfort.

She was initially hospitalized under a diagnosis of altered mental status with the suspicion of metabolic encephalopathy or central nervous system (CNS) infection. On admission, the patient was dehydrated with tachycardia. Her Glasgow Coma Scale (GCS) score for consciousness was E3V4M5. No other sign of cranial nerve dysfunction or decreased muscle power was noted and the remainder of the physical examination was unremarkable.

Laboratory investigation including blood counts, electrolytes, and renal and liver function test were normal except for hemoconcentration (blood urea nitrogen, 26.7 mg/dL; serum creatinine, 1.52 mg/dL; Na+, 153 mmol/L). To evaluate the causes of altered mental status, lumbar puncture was performed with the following results: cell count, 0/cumm; glucose, 100 mg/dL; polymerase chain reaction (PCR) for herpes simplex virus (HSV), negative. Cerebrospinal fluid (CSF) culture for bacteria, *Mycobacterium*, fungi and virus proved to be negative. Brain contrast-enhanced magnetic resonance imaging revealed senile brain atrophy without evidence of encephalitis or meningeal inflammation.

**Fig. 1:**
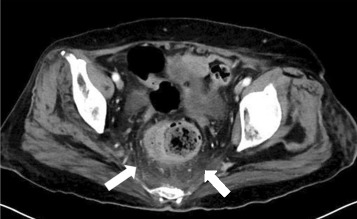
Abdominal contrast-enhanced computed tomography (CT) and histopathology. Abdominal contrast-enhanced CT on day 10 of admission revealed increased inflammation surrounding the rectum, suggestive of proctitis (*arrow*)

Under a presumptive diagnosis of metabolic encephalopathy or CNS infection, acyclovir and ceftriaxone were prescribed for 5 days until negative CSF results (bacterial culture and HSV PCR) were confirmed. The patient’s consciousness level returned rapidly to GCS E4V5M6 after fluid supplementation for initial dehydration. However, she subsequently presented with abdominal pain and diarrhea on day 8 of admission. We initially treated the patient for nosocomial diarrhea, including adjusting her enteral feeding diet and medication, and surveying the stool for *Clostridium difficile* infection. However, stool analysis disclosed negativity for fecal white blood cells and *C. difficile* toxins A and B, but positivity for occult blood. Because of persistent GI discomfort, abdominal contrast-enhanced computed tomography was performed on day 10 of admission, which demonstrated inflammation around the rectum, suggesting proctitis (Fig. 1).

**Fig. 2:**
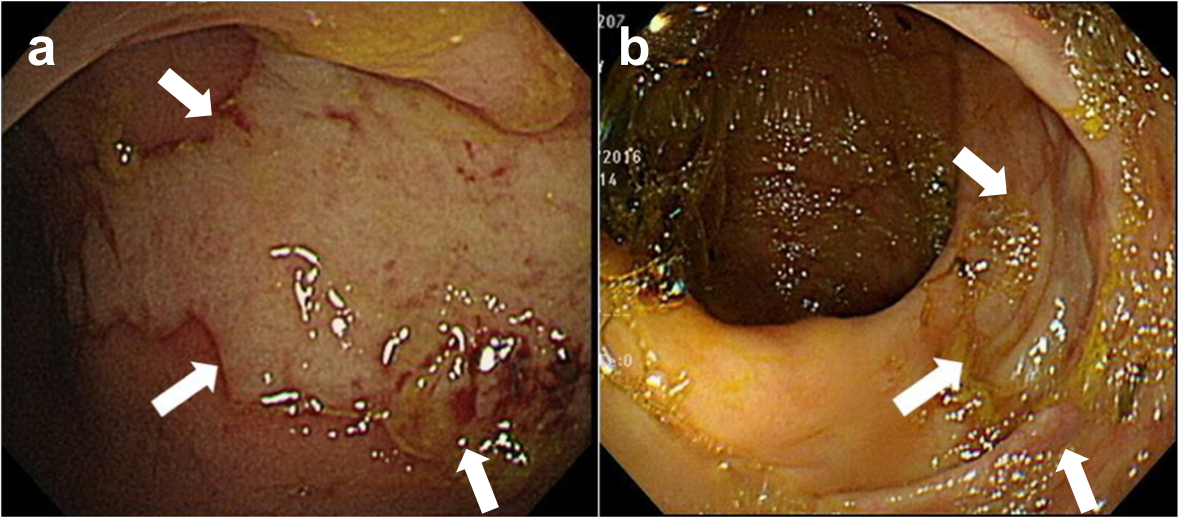
Colonoscopy before and after antiviral therapy. **a** Colonoscopy on day 16 of admission revealed a giant ulcer with mucosal coating at the rectum (*arrow*). **b** Colonoscopy after 38 days of valganciclovir treatment revealed a healed ulcer over the rectum (*arrow*)

**Fig. 3:**
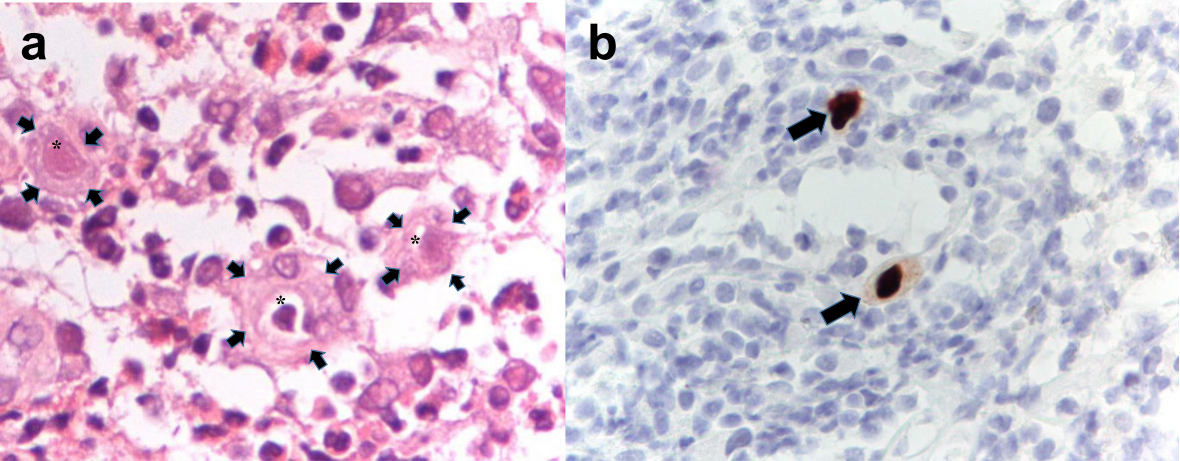
Microscopic examination of rectal biopsy. **a** Hematoxylin and eosin staining (x 400) revealed ulcerative colonic mucosa with granulation tissue and scattered atypical cells (*arrow*) bearing basophilic intranuclear inclusion bodies with perinuclear halo (asterisk). **b** Immunohistochemical staining with monoclonal antibodies for CMV (x 400) demonstrated positive uptake in inclusion bodies (*arrow*)

Colonoscopy was performed on day 16 of admission, which showed a giant ulcer with mucus coating at the rectum (Fig. 2a). Pathology of a rectal biopsy at 10 cm above the anal verge confirmed CMV proctitis (Fig. 3a and b). Serological tests to detect CMV immunoglobulin (Ig) G and IgM antibodies were positive and negative, respectively. HIV enzyme-linked immunosorbent assay and a CMV pp65 antigen test were both negative.

The patient received 450 mg of valganciclovir twice daily for 38 days and her intestinal discomfort, including abdominal pain and diarrhea, improved completely. Follow-up colonoscopy performed after 38 days of valganciclovir treatment revealed a healed ulcer over the rectum (Fig. 2b).

## Discussion

We performed a literature search of articles published from 1980 to 2015 using the PubMed database with the search terms “CMV proctitis,” “cytomegalovirus,” and “proctitis”. References from relevant articles were also reviewed for potential cases. Only cases of reactivated CMV proctitis in immunocompetent patients were included. Although reactivation of CMV frequently occurs in aging, it is subclinical in the majority [6]. Therefore, although elderly individuals are less immunocompetent than young population [6], they are not considered to be profoundly immunocompromised in our literature view. Immunocompetent patients were defined as patients without profound loss of immune function, thus we excluded AIDS patients, solid organ or hematopoietic stem cell transplant recipients, or patients receiving immunosuppressive agents. A total of 11 cases (including the present case) of reactivated CMV proctitis in immunocompetent patients were identified from five published articles. Comorbidities, clinical manifestation, endoscopic findings, therapeutic interventions and outcomes were analyzed (Table 1).

**Table 1:** Comorbidities, clinical characteristics, and outcomes of 11 cases of CMV proctitis in immunocompetent patients

Case, Reference, Publication year	Sex/Age (yr)	Comorbidities	Presenting Symptoms	Preceding condition	Endoscopic finding	Pathological finding^a^	Treatment	Outcome
1 [21], 2007	M/71	DM, arrhythmia	Hematochezia	Esophageal adenocarcinoma with esophagectomy complicated with multiple organ failure	Multiple ulcers	Yes	Proctectomy and colostomy	Died (due to massive rectal bleeding)
2 [22], 2006	F/86	N/A	Anorexia, diarrhea	N/A	Two fistulas (into the vagina and urinary bladder)	Yes	Ganciclovir	Improved
3 [23], 1999	F/57	DM	Diarrhea, tenesmus, and difficulty in defecating	*Klebsiella pneumoniae* bacteremia complicated with disseminated hepatic microabscesses and septic pneumonia	Large ulceration with multiple fistulas and sinus tracts	Yes	Ganciclovir	Improved
4 [12], 1999	F/83	DM, stroke	Bloody diarrhea	Pseudomonas pyelonephritis	Rectal ulcer	Yes	Supportive	Improved
5 [12], 1999	F/59	Hypertension, CKD	Bloody diarrhea (5 days after MI)	Acute MI with cardiogenic shock	Rectal erosions	Yes	Supportive	Died (due to heart and renal failure)
6 [12], 1999	M/63	IHD, CKD	Diarrhea, fever (12 days after MI)	Acute MI, stroke	Rectal erythema	Yes	Supportive	Died (due to pneumonia)
7 [12], 1999	F/69	DM	Hematochezia, fever	Hyperosmolar diabetic coma, pneumonia, and cystitis	Circumferential ulcer at the rectum	Yes	Supportive	Died (due to pyelonephritis)
8 [12], 1999	F/92	Meningioma; chairbound	Bloody diarrhea, fever	Cholangitis	Rectal ulcer	Yes	Ganciclovir	Rectal stricture, improved
9 [12], 1999	F/74	Osteoporosis, DM, parkinsonism	Bloody diarrhea	N/A	Sessile growth at rectum	Yes	Ganciclovir	Died, (due to septic shock; had a rectovaginal fistula)
10 [24], 1988	M/65	N/A	Hematochezia	Motorcycle accident	Rectal erythema with a polypoid mass and punch-out ulcers	Yes	Sulfasalazine	Improved
11, current case	F/79	DM. IHD, major depression disorder	Abdominal pain, diarrhea, tenesmus	Metabolic encephalopathy, delirium	Rectal ulcer	Yes	Valganciclovir	Improved

Abbreviations: *CKD* chronic kidney disease, *DM* diabetes mellitus, *F* female, *IHD* ischemic heart disease, *M* male, *MI* myocardial infarction, *N/A* not available

^a^Includes compatible CMV inclusion bodies on pathology

In our review of 11 cases, the mean age was 72 years (range, 57–92 years); three (27.3%) were male; six (54.5%) had DM; eight (72.7%) initially presented with preceding conditions unrelated to CMV proctitis; five (45.5%) died. Among the 11 cases, five patients (45.5%) received ganciclovir or valganciclovir, and local complications related to proctitis developed in five patients (45.5%) (massive rectal bleeding, *n* = 1; rectal fistula to adjacent organs, *n* = 3; rectal stricture, *n* = 1).

In our review, 72.7% of CMV proctitis cases occurred during hospitalization for a preceding condition, rather than as an initial presentation on admission. Cardinal manifestations are often non-specific (e.g., diarrhea, hematochezia, tenesmus), and these presentations, while developing during hospitalization, are often regarded as nosocomial diarrhea. Nosocomial diarrhea, however, is a common complication of hospitalization that is generally caused by non-infectious etiologies, such as medications and enteral feeding. *C. difficile* is the most common infectious etiology of nosocomial diarrhea [4], while CMV as an etiology of nosocomial diarrhea is reported mainly in transplant patients [4, 5]. Based on our findings, therefore, CMV proctitis should also be included in the differential diagnosis in a staged approach of diagnosing nosocomial diarrhea in hospitalized adults, especially for aged patients with DM.

We found that 72.7% of patients developed reactivated CMV proctitis following a preceding acute, life-threatening disease. This finding, however, was also observed in previous reports of CMV colitis in immunocompetent patients [7, 8]. In a previous review, CMV diseases were reported in 0–36% of critically ill immunocompetent patients, mostly developing 4–12 days after ICU admission [9]. Disruption of the colonic mucosa caused by preceding conditions, such as medication, inflammatory bowel disease, radiation therapy, and cardiogenic shock, may result in CMV reactivation [8–10]. Sepsis may also lead to CMV reactivation through the inability of interleukin-2 to restore natural killer cell function, which is important in controlling the virus after acute infection and reactivation [11]. Complex interactions among the inflammatory cascade, the reactivation stimulus, and viral load on the pathogenesis of CMV reactivation in immunocompetent patients require further investigation [11].

CMV proctitis and colitis may not be regarded as two distinctive disease entities because these two sites of involvement often occur concomitantly [2, 10, 12, 13]. However, compared with previous studies of CMV colitis in immunocompetent patients by Polymnia et al. (mean age, 61.1 years; DM, 13.6%) [14] and Karakozis et al. (mean age, 63 years) [15], the 11 cases in our review appeared to be older (mean age, 72 years) and had a higher prevalence of DM (54.5%). These findings imply that immunocompetent patients with more vulnerable factors affecting immune responses are more prone to CMV proctitis with or without colitis than to CMV colitis alone.

The significance of negative CMV pp65 antigenemia in the present case with confirmed CMV proctitis remains unknown. CMV pp65 antigen in polymorphonuclear leukocytes is used to detect CMV viremia. There have been several studies to evaluate the utility of CMV pp65 antigenemia as an indicator for pre-emptive therapy in the immunocompromised host, such as transplant and AIDS patients [16, 17]. The cut-off for CMV pp65 antigenemia to start pre-emptive therapy varies among different patient populations [16], but the relevant threshold in the immunocompetent host is lacking. Besides, it is not uncommon to observe negative CMV pp65 antigenemia in immunocompromised patients with confirmed CMV disease [18], and the relevant data about the prevalence of negative CMV pp65 antigenemia in immunocompetent hosts with confirmed CMV disease is also absent. In summary, the utility of CMV pp65 antigen testing in immunocompetent hosts as an indicator for pre-emptive therapy or diagnosis of CMV disease requires further investigation.

In our review, one of five patients receiving antiviral therapy died (related to local complications of proctitis); and four of six patients without antiviral therapy died (one case related to CMV proctitis). Though there is a difference in the overall mortality rate between those with and without antiviral treatment, unlike the definitive role of antiviral therapy in the management of CMV disease in immunocompromised patients [1, 19, 20], no clear conclusion can be drawn about the benefit of antiviral therapy for CMV proctitis in immunocompetent patients based on this small, non-controlled study. A previous review of severe CMV infection in immunocompetent patients also revealed inconclusive results [2]. The improvement observed in some of the patients receiving antiviral agents may be related to the typically self-limiting course of CMV disease in immunocompetent hosts, rather than to any antiviral effect [2]. Considering the rarity of reactivated CMV disease in immunocompetent patients, it is difficult to conduct a randomized controlled trial. Therefore, in immunocompetent patients, the potential benefit of antiviral treatment should be cautiously weighed against the potential toxicity of antiviral therapy.

## Conclusions

In conclusion, reactivated CMV proctitis in immunocompetent patients is rare, and has been reported mainly in elderly patients with comorbidities, especially DM. We believe reactivated CMV proctitis is an important, but underestimated etiology of nosocomial diarrhea. Clinicians should be aware of the possibility of nosocomial onset of reactivated CMV proctitis in patients hospitalized due to a preceding, critical illness, although the benefits of antiviral therapy remain unclear.

## Abbreviations

AIDS: Acquired immunodeficiency syndrome
CMV: Cytomegalovirus
CNS: Central nervous system
CSF: Cerebrospinal fluid
DM: Diabetes mellitus
GCS: Glasgow Coma Scale
GI: Gastrointestinal
HIV: Human immunodeficiency virus
HSV: Herpes simplex virus
ICU: Intensive care unit
Ig: Immunoglobulin
PCR: Polymerase chain reaction
